# Belladonna in Opium Poisoning

**Published:** 1874-10

**Authors:** James O. Lewis

**Affiliations:** Greenwood, Fla.


### BELLADONNA IN OPIUM POISONING.

By JAMES O. LEWIS, M.D., Greenwood, Fla.

*Editor Atlanta Medical and Surgical Journal:*

Allow me space in your Journal for the following case, which I think of importance, in showing the antagonistic properties of belladonna to morphine:

On August 1st was called in consultation with Dr. Abernathy to see a little son of Gen. Barnes, age 18 months. Found him with cool skin, eyes closed, and in almost complete coma. So much so that it was with great difficulty I could get any manifestations of life; breathing slow and labored, pupil a mere point. Was informed he had taken one full grain of sulph. morphine 24 hours before; it was then 7 o'clock A.M. His pulse was very frequent and feeble. I learned an effort had been made, about five or six hours after the morphine was taken, to vomit him, but failed. Dr. A. had kept him awake as much as possible, and thinking him better, the same evening allowed him to sleep, when he grew so much worse that I was called in. When I arrived I think they were rubbing him with mustard. I immediately commenced the use of alc. extract of belladonna, in grain doses every hour, and had the satisfaction to see it exert its usual power of dilating the pupils. At the same time the breathing grew better, he was more easily aroused, and by 11 o'clock I pronounced him out of danger, and at 2 P.M. allowed twenty minutes sleep, when he awoke all right. Among other things done, I ordered a mustard over upper part of spine, and allowed it to redden the parts well. I wished to arouse the medulla as much as possible. I procured a small magnetic instrument, with which I sent a current from base of brain to ensiform cartilage, or pit of stomach, and along the diaphram, so as to arouse the nerves of respiration and stimulate the muscles. I also passed the current through the head from front to back, and transversely too. That was about 11 o'clock, at the time the belladonna had shown its power, and I did not think electricity or any other agent really necessary to carry my little unfortunate through. I will not omit a plentiful supply of strong coffee and milk punch, which were used as freely as we thought prudent. How would bromide of potassium do in such cases? It is generally believed to lessen the quantity of blood in the brain; so it would seem to be the indication. I gave the little boy about three grains of the extract of belladonna before I was satisfied he was safe.

